# Decoding fairness motivations from multivariate brain activity patterns

**DOI:** 10.1093/scan/nsz097

**Published:** 2020-01-09

**Authors:** Sebastian P H Speer, Maarten A S Boksem

**Affiliations:** Rotterdam School of Management, Erasmus University, 3062 Rotterdam, The Netherlands

**Keywords:** prosocial behavior, theory of mind, cognitive control, fMRI, machine learning

## Abstract

A preference for fairness may originate from prosocial or strategic motivations: we may wish to improve others’ well-being or avoid the repercussions of selfish behavior. Here, we used functional magnetic resonance imaging to identify neural patterns that dissociate these two motivations. Participants played both the ultimatum and dictator game (UG–DG) as proposers. Because responders can reject the offer in the UG, but not the DG, offers and neural patterns between the games should differ for strategic players but not prosocial players. Using multivariate pattern analysis, we found that the decoding accuracy of neural patterns associated with UG and DG decisions correlated significantly with differences in offers between games in regions associated with theory of mind (ToM), such as the temporoparietal junction, and cognitive control, such as the dorsolateral prefrontal cortex and inferior frontal cortex. We conclude that individual differences in prosocial behavior may be driven by variations in the degree to which self-control and ToM processes are engaged during decision-making such that the extent to which these processes are engaged is indicative of either selfish or prosocial motivations.

## Introduction

Our social life abounds with situations in which there is a conflict between selfish urges and the welfare of others. An example would be whether to donate a recently received windfall to charity or spend it on a new phone. Whereas highly prosocial individuals such as Martin Luther King or Mother Theresa may devote their life to improve the condition of the oppressed or the less fortunate, others tend to focus on maximizing their own gains and in some cases even exploit others. These large differences in how individuals weigh their own benefits against another person’s welfare are crucially important in understanding social decision-making that enables cooperation on a societal level. Yet, until now the underlying psychological and neural mechanisms of these individual differences in prosociality have remained largely elusive.

A common framework to study the trade-off between prosociality and selfishness is the ultimatum game (UG) (Güth *et al.*, 1982). In the UG a proposer can decide how to divide a sum of money, and a recipient can subsequently accept or reject this proposal. In case the recipient accepts the offer, the money is distributed as proposed, whereas nobody receives anything in case the responder rejects the offer. Previous research shows that the majority of proposers split the money about equally and offer on average 40% of the initial endowment (Oosterbeek *et al.,* 2004; Henrich *et al.,* 2005). In order to explain why proposers deviate from purely selfish behavior, two competing mechanisms have been proposed. Proposers may decide to share because they care for the welfare of their opponent and may thus be driven by prosocial or fairness concerns (Thaler, 1988; Oosterbeek *et al.,* 2004). Alternatively, proposers may give high offers to reduce the chance of rejection and strategically maximize their financial gains, reflecting essentially selfish motives (Forsythe *et al.,* 1994; Fehr and Schmidt, 1999).

To test which of these mechanisms dominates, the UG can be used in combination with the dictator game (DG), in which the responders cannot reject the offers. Hence, there is no punishment threat in place to prevent selfish behavior. As a consequence, the proposers’ decisions in the DG are considered a straightforward reflection of their social preferences, as they are uncontaminated by strategic considerations. Critically, proposers motivated by prosocial fairness concerns should offer equally in both games as the importance of welfare of the opponent is unchanged across games, while selfish proposers are expected to strategically split their endowment equally in the UG to reduce the chance of rejection and give low offers in the DG.

Accumulative evidence from previous studies indicated that while the average offers in the DG are significantly lower than in the UG, they still remain well above what would be expected by standard economic theory (Kahneman *et al.,* 1986; Forsythe *et al.,* 1994). These findings suggest that some proposers indeed change the size of their offer quite drastically between games, while others offer similar amounts in both games. Thus, based on high *vs* low differences in offers between games, we can distinguish between those who are essentially selfish and only strategically increase their offer when punishment is possible, whom we characterize as selfish players, and those who do not, characterized as prosocial players.

Previous neuroimaging research on the proposers’ motivations in the UG and DG has focused particularly on social norm compliance, converging on the crucial involvement of the dorsolateral prefrontal cortex (dlPFC) and cognitive control in strategic fairness in the UG (Yamagishi *et al.,* 2016; Strang *et al.,* 2014; Spitzer *et al.,* 2007; Steinbeis *et al.*, 2012). The experimental evidence from these studies generally suggests that in order to act prosocially in the UG, proposers need to control selfish impulses, which is reflected in increased dlPFC activity.

Other studies, however, have challenged this central role of cognitive control in prosocial behavior. Evidence comes from two meta-analyses indicating that prosocial choices and cooperation across various different economic games, such as the trust game, public goods game and DG are more frequent when cognitive control is reduced, such as when one is under time pressure (Rand *et al.,* 2015; Rand, 2016) and when primed to trust ones intuitions (Rand *et al.,* 2012; Lotz, 2015). Furthermore, it has been shown that when cognitive control is interfered with, for example, by concurrently performing an *N*-back task, proposers made more generous offers in the DG (Schulz *et al.,* 2014). Collectively, these results suggest that, at least under some conditions, self-control may not be necessary to act prosocially, and reduced self-control may even promote prosocial behavior.

In a largely separate literature, it is widely accepted that theory of mind (ToM) is a strong determinant of prosocial sharing (e.g. Eisenberg and Miller, 1987; Batson *et al.,* 1991; Penner *et al.,* 2005; Pavey *et al.,* 2012; Edele *et al.,* 2013; Artinger *et al.,* 2014). For example, when proposers were instructed to imagine being in the position of a responder, they offered significantly higher amounts of money (Hoffman *et al.,* 2000).

Thus, there seems to be a disconnect between behavioral research on the proposers in the UG, emphasizing the role of ToM in prosocial behavior, and the neuroimaging literature on the proposers motivations and behavior, focusing on the role of self-control in social norm compliance. These neuroimaging studies do not discuss the relevance of ToM and do not report activity in the neural network underlying ToM: a widely distributed network of brain regions including the temporoparietal junction (TPJ), the precuneus, the medial prefrontal cortex (MPFC), the angular gyrus and the temporal lobes (Gallagher and Frith, 2003; Ciaramidaro *et al.,* 2007; Schurz *et al.,* 2014).

Here, we try to bridge this disconnect by specifically testing for differences in the ToM network in participants playing the UG. Specifically, we combined a mixed UG–DG paradigm with functional magnetic resonance imaging (fMRI) to identify neural patterns that dissociate selfish from more prosocial individuals, using multivariate pattern analysis (MVPA), which may be more sensitive to pick up more subtle differences in psychological processes such as empathy and ToM (Kriegeskorte *et al.,* 2006; Norman *et al.,* 2006).

## Methods

### Participants

The reported analyses are based on 31 participants (22 females; age 18–44 years; *M* = 24.2, s.d. = 6.2) from two separate studies. The reason for running two studies was driven by funding issues. We ran out of funding half-way through the data collection and once we obtained additional funding continued with the data collection. The first sample of participants consisted of a student sample (*N* = 19, 13 females; age 18–44 years; *M* = 23.3, s.d. = 7.0) from now on referred to as Study 1. The second sample also consisted of a student sample but from a different university (*N* = 12, 9 females; age 22–31 years; *M* = 26.2, s.d. = 2.7) from now on referred to as Study 2. No significant differences in demographics were identified between samples. All participants were right-handed with normal or corrected to normal vision and no record of neurological or psychiatric diseases. The studies were approved by the university ethics committees and were conducted according to the Declaration of Helsinki.

### FMRI acquisition

For Study 1, the functional magnetic resonance images were obtained using a 3 T Siemens Allegra MRI system. Functional scans were acquired by a *T*_2_*-weighted gradient-echo, echo-planar pulse sequence in ascending interleaved order (3.5 mm slice thickness, 3.5 × 3.5 mm in-plane resolution, 64 × 64 voxels per slice, flip angle = 90°, FOV = 224). Echo time (TE) was 30 ms and repetition time (TR) was 2000 ms. A *T*_1_-weighted image was acquired for anatomical reference (1.0 × 1.0 × 1.0 mm resolution, 192 sagittal slices, flip angle = 9°, TE = 2.6 ms, TR = 2250 ms). For Study 2, the functional magnetic resonance images were collected using a 3 T Siemens Verio MRI system. Functional scans were acquired by a *T*_2_*-weighted gradient-echo, echo-planar pulse sequence in descending interleaved order (3.0 mm slice thickness, 3.0 × 3.0 mm in-plane resolution, 64 × 64 voxels per slice, flip angle = 75°). TE was 30 ns and TR was 2030 ms. A *T*_1_-weighted image was acquired for anatomical reference (1.0 × 0.5 × 0.5 mm resolution, 192 sagittal slices, flip angle = 9°, TE = 2.26 ms, TR = 1900 ms).

### Experimental task and procedures

Participants played a mixed UG/DG in the MRI scanner. On 24 trials, participants received €20 and had to decide how to split the endowment between themselves and an opponent. On each trial they were presented with a picture of the opponent before and during the decision process, in order to ensure that participants knew they were playing against a different human player on each trial. The pictures used in the study were obtained from the NimStim face database (Tottenham *et al.*, 2009). We selected faces which were categorized as neutral and most representative of our participant population in terms of age and ethnicity. This was done to minimize the effect of the difference in faces on offers made in the games. Further choosing a representative sample of pictures was intended to increase the credibility of our cover story that participants played against previous participants. On half of the trials, the opponents were able to reject the offer, which would result in both the participant and opponent receiving nothing (UG). On the other half of the trials, the opponents were passive recipients and could not reject the offer (DG). The critical difference between these conditions is that in the UG trials, the participant can be punished for unfair offers, whereas in the DG trials no punishment is possible. In the non-social control condition (24 trials), participants played against a computer algorithm, allegedly programmed to mimic human behavior. Again, half of the trials in the control condition were UG trials and the other half DG trials. Practice trials were implemented to in order to familiarize the participants with the task. In addition, participants were told that they were playing against participants who had previously participated in the study. As mentioned above, pictures of opponents were chosen with the aim of maximizing representativeness of the sample used in order to increase credibility of the story.

**Fig. 1:**
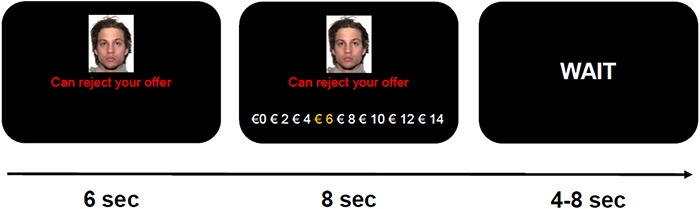
The timing of the UG task. The experiment starts with a waiting period of 8 s, followed by a decision phase of 6 s in which all the information relevant to the decision-maker is present and ends with the response phase of 8 s in which participants could indicate their choice. For the fMRI analysis, the period 6 s prior to the button press was used.

The trials started with a screen that presented a picture of the opponent and their power to reject the offer or not (UG or DG). Subsequently, the response options appeared, 0 to 14€ in steps of two, and participants could indicate their choice. Lastly, a wait screen appeared for 8 s (see Figure 1).

### fMRI analysis

#### Pre-processing

All fMRI data underwent the standard FSL (5.0) pre-processing pipeline. Anatomical scans were reoriented to the FSL standard orientation and skull-stripped. The functional data was motion corrected to the mean image using FSL’s MCFLIRT and coregistered to the anatomical scan and normalized to the standard MNI brain using boundary-based registration (FSL FLIRT and FNIRT). Subsequently, Gaussian high-pass filtering with 100 s FWHM was applied.

To obtain neural activation patterns for multivariate analysis, individual time series were modeled using a double γ hemodynamic response function (HRF) in a single-trial GLM design using FSL’s FEAT. Specifically, one GLM fitted a HRF for each trial, following the least-squares all approach (Mumford *et al.,* 2012), using the 6 s prior to the keypress in each trial, resulting in 48 (12 trials * 4 conditions) parameter estimates of sustained activations for each participant. Specifically, there were 12 trials of UG and DG in the social condition (human opponent) and 12 trials of both games in the non-social condition (computer opponent). The resulting β-values were converted to *t*-values (Misaki *et al.,* 2010), resulting in a whole-brain pattern of *t*-values for each trial. The duration of the epoch we used for our fMRI analysis was 6 s, and onset times were determined by counting back 6 s from the point in time when the participant had indicated his choice. This window was used as it provides all the necessary information to make the decision and is free of brain activity related to motor responses. Average background signal and white matter signal were entered as regressors of no interest. All regressors were convolved with the canonical HRF. In order to test whether differences between games can be decoded from the brain, we applied a classification analysis to the whole-brain activity patterns estimated from our single-trial GLM. Classification analyses were conducted with the PyMVPA toolbox (Hanke *et al.,* 2009) and custom Python scripts, which are available on figshare 10.25397/eur.11294645.

#### Relating classification accuracy to behavior in the ToM and cognitive control network

**Fig. 2:**
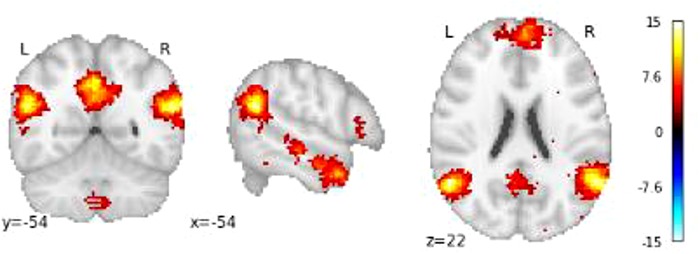
The ToM mask obtained from the meta-analysis on neurosynth showing clusters in the precuneus left and right TPJ, temporal pole and the MPFC.

**Fig. 3:**
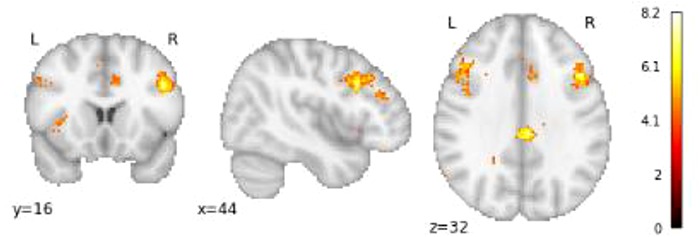
The cognitive control mask obtained from the meta-analysis on neurosynth showing clusters in the left and right dlPFC and the PCC.

First, we investigated whether neural patterns in regions associated with ToM and cognitive control processes distinguish between prosocial and selfish players. In order to do so, we obtained a ToM–brain–activation mask and a cognitive control-brain activation mask by conducting a meta-analysis via Neurosynth (Wager *et al.*, 2011, see Supplementary Appendix 1). The masks were derived from a meta-analysis of previous studies reporting brain regions that are consistently active in articles that include the term ‘ToM’ and ‘cognitive control’ in the abstract (*N* = 181 and 598, respectively; see Figures 2 and 3). Using this large-scale automated meta-analysis increases confidence that neural activation in these regions indeed reflect engagement on the hypothesized processes while reducing problems with reverse inference (Poldrack, 2006).

We extracted the activation patterns within the ToM and cognitive control mask in native space, corresponding to one decision phase each, which were labeled according to their experimental condition, either UG trial or DG. Only activation patterns for the social condition (human opponents) were selected for this analysis. Subsequently, for each activation pattern, each voxel was standardized such that they have zero mean and unit variance. Lastly, we applied univariate feature selection, in which the 1000 voxels with the highest F-value resulting from an ANOVA on the contrasts of interest (UG *vs* DG) were selected from within the masks. Subsequently, a multivariate pattern classification using a support vector machine (*C* = 1) was applied to the selected voxels (Cox and Savoy, 2003; Mitchell *et al.,* 2004). To account for possible effects of the size of the masks, 1000 voxels were selected for both masks. In order to avoid overfitting and inflated prediction accuracy (Vul *et al.,* 2009), the three steps (feature scaling, feature selection and classification) were implemented using 6-fold cross validation.

To explore how differences in neural patterns between conditions (UG *vs* DG) across participants relate to differences in behavior, we correlated the classification accuracies for each participant, derived from the above classification analysis, with the average difference in offers between games of each participant, while controlling for cohort effects. This partial correlation with a dummy variable for the cohort was used to account for possible variance introduced by the difference between the two samples and scanners used. The same analysis was conducted for activation patterns for the non-social condition in order to test whether possible effects are specific to the social condition or generalize across all trials.

#### Relating classification accuracy to behavior in individual regions in the ToM and cognitive control network

Next, we explored whether neural patterns in individual regions within the ToM and cognitive control networks differentiate between prosocial and selfish players using the same masks as above. Individual clusters within the masks were extracted and converted to each subject’s native space (see Supplementary Appendix 1). We then extracted the activation patterns within these Regions of Interest (ROIs). As before, only activation patterns for the social condition (human opponents) were selected for this analysis. Apart from the feature selection, which in this analysis was done by means of selecting individual ROIs, we applied the same analysis procedure as above.

#### Localizing other areas that discriminate between prosocial and selfish players

To explore whether activity patterns in regions outside of the ToM and cognitive control networks correlated with individual differences in behavior, a classification searchlight approach was used. More specifically, we investigated where in the brain average UG and DG offers between subjects correlate with classification accuracy between UG and DG trials. To this end, we employed a spherical searchlight of 3-voxel radius. For this analysis, the individual *t*-stat maps were smoothed (fwhm = 8) to render the output of the searchlight more clustered and more interpretable. At each location, for all voxels included in the current sphere, the searchlight performed a classification of game type using a support vector classifier implemented with 6-fold cross validation. This was done for all subjects, resulting in 31 classification maps, where each voxel represents the classification accuracy of local UG *vs* DG patterns. Subsequently, to test whether within-subject classification accuracy between UG and DG correlates with individual differences in behavior, we calculated at each voxel a Pearson’s correlation between the classification accuracy and mean difference in offers between UG and DG trials while partialing out the group membership to the two cohorts (Study 1 and Study 2). We estimated statistical significance by permutation testing. Specifically, we shuffled the differences in behavior 5000 times and obtained null correlation maps and derived the empirical *p* value at each voxel from the voxel’s own null distribution.

## Results

### Behavioral results

#### Social trials

**Fig. 4:**
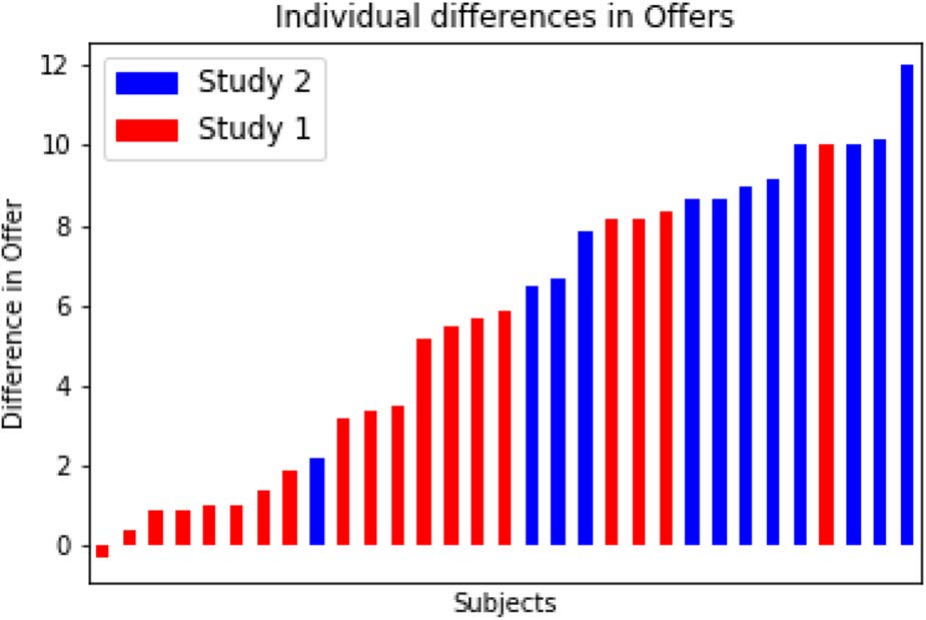
Differences in offers (UG–DG) between games for Human trials across participants in both studies.

On average, participants made higher offers in the UG than in the DG, but this difference varied substantially between subjects (UG–DG; *M* = 8.65, s.d. = 3.27; see Figure 4): some participants offered much more in the UG, while other subjects made very similar offers in both games. The lowest mean offer observed on a UG trial for a participant was €8.5 which excludes the possibility that low differences in offers between games could be due to low offers in both games (see Supplementary Appendix 2, Figure S1). From now on we refer to participants with a low difference in offers between games as prosocial players (as they give high offers in both games), whereas participants that exhibited high difference in offers between games as selfish players (as they only strategically offer high amounts when punishment is possible but give low offers otherwise).

**Fig. 5:**
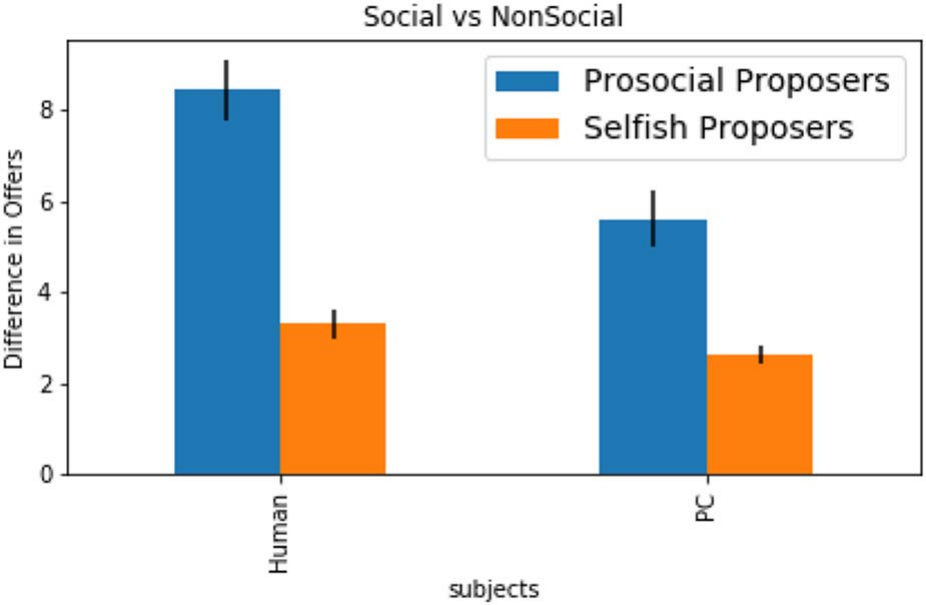
Bar graph illustrating the difference in offers in the DG between prosocial and selfish proposers (median split, for illustrative purposes only) on human and computer trials.

As this study contained data from two different samples using different scanners, we tested whether there were differences in behavior between the two samples using a two-sample *t*-test and found that the differences in offers between games was significantly higher in the second sample as compared to the first sample (*t*(df = 29) = −4.41, *P* < 0.001), which, in addition to the different scanning parameters used in the two studies, necessitated using partial correlation for our fMRI analyses.

#### Behavioral results for social vs non-social trials

Here, we investigated whether participants give high offers out of *social* motivations (compliance with social norms or prosocial behavior) or whether they are driven by a more general response to any type of social or non-social punishment threat. If the underlying decision-making processes are indeed social, we would expect prosocial participants to exhibit larger differences in offers between the social and non-social condition as compared to the more strategic players. To test this, a multiple regression analysis was performed to predict offers in the DG based on the type of opponent (human *vs* computer) as a dummy variable, the mean difference in offer between games as continuous regressor (indicating the extent to which participants are prosocial or selfish) and the interaction between these two variables. Only DG trials were used as differences between motivations (social *vs* non-social and fair *vs* selfish) which should be expressed particularly on these trials. The regression model was found to be significant (*F*(2.58) = 67.78, *P* < 0.001), with an *R^2^* of 0.882. Participants gave higher offers in the social condition than in the non-social condition (*b* = 3.725, SE = 0.66, *t* = 5.63, *P* ≤ 0.001), and DG offers decreased with higher difference in offers between games (more selfish participants offered less; *b* = −0.461, SE = 0.07, *t* = −6.585, *P* ≤ 0.001). Importantly, a significant interaction effect was found between the difference in offers (prosocial *vs* selfish players) and type of opponent (human *vs* computer), *b* = −0.358, SE = 0.099, *t* = −3.618, *P* ≤ 0.001: in the social condition participants made significantly higher DG offers than in the non-social condition and that this effect was stronger for prosocial players as compared to selfish players (see Figure 5).

### fMRI results

#### Classification accuracy of game type correlates with prosociality in the ToM and cognitive control networks

**Fig. 6:**
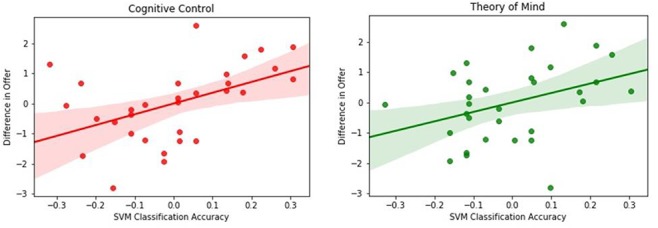
Correlation between classification accuracy and difference in offer (UG–DG) for neural patterns in the cognitive control network (red) and the ToM network (green).

For the social trials, we found that classification accuracy in the cognitive control network significantly correlated with the difference in offers between games (*r* = 0.48, *P* < 0.01). Further, classification accuracy on patterns from the ToM network also significantly correlated with difference in offers (*r* = 0.37, *P* < 0.05). This suggests that neural patterns in both the cognitive control network and the ToM network differentiate between prosocial and selfish players (see Figure 6). For the non-social trials, no significant correlation was found for the ToM network (*r* = 0.18, *P* = 0.34) or the cognitive control network (*r* = 0.30, *P* = 0.09).

**Fig. 7:**
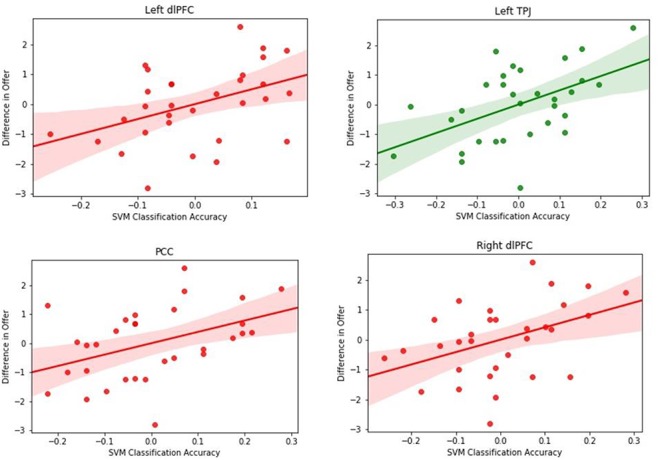
Correlation between classification of game type accuracy in the right dlPFC, left TPJ, PCC and left dlPFC and average difference in offer (UG–DG) between games across participants while controlling for difference in cohorts.

**Fig. 8:**
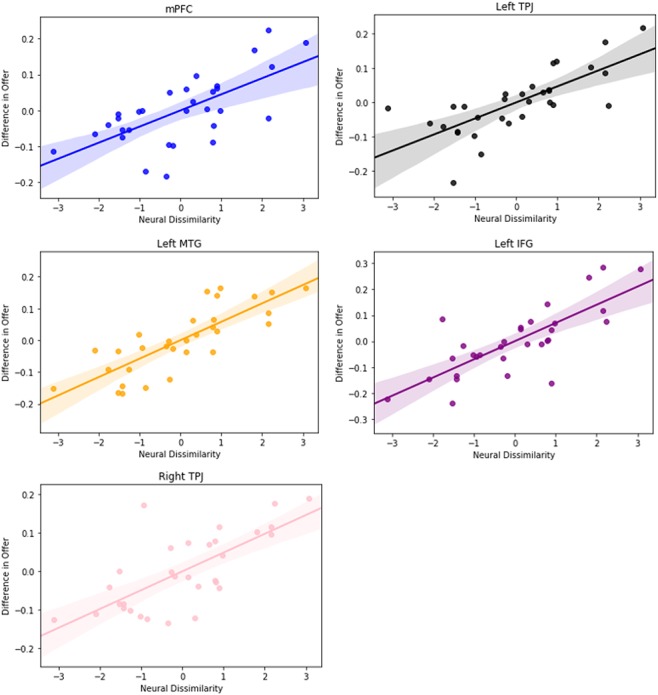
The correlations between classification accuracy and the average difference in offers (UG–DG) between games in the MPFC (blue), the left TPJ (black), the left MTG (yellow), the left IFG (purple) and the right TPJ (pink).

#### Classification accuracy correlates with prosociality in specific regions within the cognitive control and ToM network

##### ToM network

When correlating the individual differences in offers between games with the classification accuracy from the support vector classifiers, we found a strong positive partial correlation in the left TPJ (*r* = 0.5, *P* < 0.005, *P*_adjusted_ = 0.03, FDR corrected for multiple tests at *P* = 0.05) (see Figure 7). Thus, neural patterns in the left TPJ differ more strongly between games for selfish players than for prosocial players. We repeated this analysis for the non-social condition and found that there were no significant correlations between the classification accuracy and differences in offers between games in any of the ROIs. Hence, the effects observed are specific to the social condition, which suggests that the differences in neural patterns observed indeed reflect social processes.

##### Cognitive control network

Within the cognitive control network, the analysis on the social trials showed that difference in offers correlated significantly with classification accuracy in the left dlPFC (*r* = 0.44, *P* = 0.01, *P*_adjusted_ = 0.03, FDR corrected at *P* = 0.05), the right dlPFC (*r* = 0.41, *P* = 0.02, *P*_adjusted_ = 0.04, FDR corrected at *P* = 0.05) and the posterior cingulate cortex (PCC; *r* = 0.46, *P* = 0.008, *P*_adjusted_ = 0.03, FDR corrected at *P* = 0.05) (see Figure 7). For the non-social trials, no significant correlations were found for any of the regions extracted from the cognitive control network. These findings indicate that neural patterns in the bilateral dlPFC and the PCC differentiate between prosocial and selfish players and that this effect is specific to the social condition. This suggests that prosocial, as opposed to selfish participants, does not need cognitive control to overcome selfish impulses and gives high offers in the social condition as they empathize with their human opponents, resulting different patterns in the cognitive control regions. However, in the non-social condition, ToM processes are not engaged, so cognitive control processes are similarly engaged for prosocial and selfish players resulting in no difference in patterns.

As the size of the ROIs (number of voxels) varied within and between the two networks, we also tested whether differences in the size of the ROIs between the two masks might have had an effect on the correlation between difference in offer and classification accuracy. We computed the correlation between these two measures and found no significant correlation (*r* = −0.25, *P* = 0.21). In addition, no significant correlation was found between classification accuracy and the size in voxels of the ROIs used (*r* = −0.04, *P* = 0.84).

#### Behavioral–neural classification searchlight

**Fig. 9:**
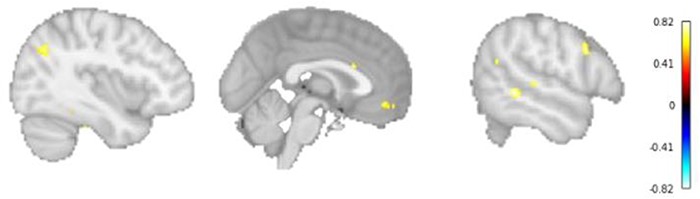
The classification searchlight analysis reveals clusters in the left TPJ (left), MPFC (middle) and right MTG and IFG (right) exhibiting significant correlation between difference in offers between UG and DG and classification accuracy on game type.

The searchlight analysis conducted on trials from the social condition revealed that classification accuracy for decoding neural patterns associated with UG and DG trials correlated significantly with individual differences in offer size between games in the left TPJ, the right TPJ, the left middle temporal gyrus (MTG), the MPFC and the left inferior frontal gyrus (IFG) (see Figure 8). For selfish players, the support vector machines were better at dissociating game type in these regions (only clusters with size of >15 voxels are reported here, see Figure 9; for all significant clusters, see Supplementary Appendix 3, Table S7). The fact that some of the regions (R TPJ and MPFC) were found to show significant correlation using the searchlight approach but not in the previous ROI analysis may be due to the higher specificity of the searchlight approach. Whereas in the ROI analysis uninformative voxels may have been included, the searchlight assesses differences in patterns in highly localized neighborhoods of a very small number of voxels. The effects observed here may be specific to only specific parts of these regions, which may also explain the small size of the clusters. In addition, a significant cluster in the right occipital cortex was found, which most likely is due to the different colors (red and blue) used to demarcate an UG or DG trial during the decision phase.

We repeated the analysis for the non-social condition and found no significant correlations in any other region (for significant clusters see Supplementary Appendix 3, Table S8). Comparing the correlations between classification accuracy and behavior in the social and non-social condition in these regions directly after performing a Fisher z transformation, we found that the correlations are indeed significantly higher in the social condition in all reported areas.

## Discussion

In order to investigate whether prosocial *vs* selfish motivations can be decoded from neural activation patterns, we conducted an experiment in which participants played the UG and DG against humans and computers while in an MRI scanner. Our behavioral results indicate that there are large individual differences in offers between the UG and DG, demonstrating that there is strong heterogeneity in motivations.

In the literature, both ToM and the cognitive control processes have been proposed to underlie individual differences in prosociality in economic exchanges. Therefore, we wanted to test whether the relationship between differences in neural patterns and behavior can be attributed to either the cognitive control or the ToM network specifically or whether both networks contribute to the individual differences in prosociality. To this end, we identified brain regions associated with ToM and cognitive control using large-scale automated meta-analysis, to increase our confidence that the selected regions indeed represent the hypothesized psychological mechanisms and thus reducing reverse inference problems (Poldrack, 2006).

Our classification analysis on the obtained ToM and cognitive control networks revealed that classification accuracy correlated significantly with difference in offers between games in both of these networks. These results indicate that neural patterns in regions in the ToM network (particularly in the TPJ) as well as in the cognitive control network (particularly in the dlPFC and PCC) appear to dissociate between prosocial and selfish players, suggesting that more selfish players engage these processes differently between games, while more prosocial players employ ToM and cognitive control similarly in both UG and DG. A whole-brain classification searchlight confirmed a significant correlation, specifically in the social condition, between neural activation patterns and classification accuracy in the bilateral TPJ but also in the MPFC and in the left MTG; all areas associated with ToM (for meta-analysis see Schurz *et al.,* 2014; Young *et al.,* 2010a).

Neuroimaging research has identified the ToM network as the basic system that facilitates social understanding (Saxe and Wexler, 2005; Schurz *et al.,* 2014; Young *et al.,* 2010b). In this network, the TPJ has not only been consistently associated with reorienting of attention and perspective taking (Gallagher and Frith, 2003; Krall *et al.,* 2015) but also with appreciating differences between one’s own and others’ perspectives in cases of conflict between those perspectives (Van Overwalle and Baetens, 2009; Hétu *et al.,* 2012). Downregulating the TPJ using TMS impaired participants’ ability to take into account another person’s intention in moral judgements (Young *et al.*, 2010a). Similarly, neuroimaging research has found the MTG to be preferentially activated for inferring others’ beliefs (Zaitchik *et al.,* 2010; Bruneau *et al.,* 2012). A meta-analysis of 40 imaging studies also found the MPFC to be a core area in social mentalizing responsible for inferring others’ dispositions in order to make accurate predictions of their next moves (see review Van Overwalle and Baetens, 2009; Schurz *et al.,* 2014). In addition, Krause and collexagues (2012) have found that for more empathic individuals, deep rTMS stimulation of the MPFC disrupted affective ToM performance, confirming the prominence of the MPFC in mentalizing processes.

In the UG and DG, the TPJ, MTG and MPFC may thus be involved in orienting the proposer’s attention to the responder and understanding her intentions and desires for both prosocial and strategic reasons. Our findings suggest that prosocial participants may have been more inclined to consider the welfare of their opponents and utilizing ToM processes equally in both games. In contrast, selfish players may have selectively and strategically engaged ToM to infer the expectations of their opponents in the UG to avoid financial punishment, whereas the opponents’ desires were irrelevant in the DG rendering inferences about their expectations redundant.

However, our findings also emphasize the involvement of cognitive control processes in social decisions. We found higher classification accuracy in the dlPFC, PCC and IFG when decoding game type for selfish players than for prosocial players. The dlPFC has been frequently implicated in cognitive control to overcome impulsive behavior (Dalwani *et al.,* 2011; Weygandt *et al.*, 2015), whereas the IFG and PCC have been consistently associated with the inhibition of predominant responses (Wager *et al.,* 2005; Verbruggen and Logan, 2008; Sharp *et al.,* 2010; Stokes *et al.,* 2011).

This suggests that prosocial participants, concerned with the welfare of their opponents, intuitively gave fair offers in both the UG and DG and did not require cognitive control or response inhibition during the decision process. Selfish players, in contrast, needed to exert cognitive control in order to overcome the selfish motivation of giving a low offer in the UG, which was necessary to avoid punishment and to maximize financial gains. Further, to inhibit this predominant selfish response and make a more generous offer, response inhibition was crucial. However, in the DG, where no punishment for selfishness was expected, the selfish player could rely on his intuitions and give low offers to maximize his monetary reward, thus not requiring cognitive control and response inhibition processes. Collectively, our findings highlight the importance of both self-control processes, reflected by activity patterns in the dlPFC, the IFG and the PCC, and the crucial involvement of empathy and ToM, represented by neural patterns in the TPJ, MPFC and the MTG.

To validate that our findings are indeed of a *social* nature and are not driven by more general processes related to response inhibition, avoidance of punishment or reward sensitivity, we ran several manipulation checks contrasting the social condition (human opponents) against our control condition in which participants were playing against a computer algorithm. We found that in the social condition, participants gave significantly higher offers than in the control condition, and this effect was significantly stronger for prosocial players as compared to players with more selfish motivations. This implies that particularly the prosocial participants care about the welfare of their human opponents; as for them the increase due to the social context is strongest. Furthermore, a supplementary univariate analysis showed that there was higher activation in the social condition than in the control condition in the MPFC, precuneus and left TPJ, which have all have been associated with empathy and perspective taking, as mentioned above (see Supplementary Appendix 4, Table S9 for list of significant clusters). In addition, when running our classification analysis on the ToM and cognitive control network, as well as on individual ROIs within those networks, no significant correlations were found between behavior and classification accuracy for the non-social condition. Collectively, these results provide evidence that participants indeed empathized with their *human* opponents and took their desires into consideration.

In the literature on individual differences in prosocial behavior during economic decision-making, there has been a disconnect between behavioral research promoting the role of ToM and empathy and most of the neuroimaging research focusing on self-control in social norm compliance. On the one hand, it has been shown that empathy is the strongest predictor of prosocial sharing (Edele *et al.,* 2013). Further, prosocial participants have been found to hold more accurate beliefs about their opponents’ offers in the UG and DG, unless these beliefs become instrumental to maximizing financial gains, in which case the selfish players perform equally well (Artinger *et al.,* 2014). On the other hand, neuroscientific evidence converges on the crucial involvement of cognitive control of the impulsive pursuit of self-interest (Strang *et al.*, 2014; Spitzer *et al.,* 2007, Steinbeis *et al.*, 2012). Further, Spitzer *et al.* (2007) showed that increased activity in the dlPFC, assumed to reflect cognitive control, was associated with higher offers in the UG.

Our findings bridge these two streams of research by highlighting the importance of both ToM and cognitive control processes. We propose that individual differences in the tendency to engage ToM may determine whether or not a person acts prosocially or selfishly. Based on this assumption, a person who intuitively considers the welfare of the opponent will not require cognitive control to make fair offers regardless of whether a punishment threat is in place or not. In contrast, an individual who is less inclined to empathize may use ToM strategically to avoid punishment and may thus require cognitive control to overcome intuitive selfish impulses. Some individuals may have a natural tendency to empathize, whereas others use empathy more strategically. In economic exchanges such as the UG and DG, this tendency for empathy may determine whether players are prosocial or follow strategic motives.

Due to the fact that multivariate neural patterns do not provide information about the strength of activation but only indicate that information is encoded differently in these regions, we cannot exclude the possibility that the effect goes in the opposite direction: prosocial proposers may have exerted cognitive control and response inhibition in both games and strategic players which may have only done so in the UG and not in the DG, which would have also resulted in higher differences in patterns in the cognitive control network for selfish players. This interpretation would suggest that all participants are intuitively selfish and that the prosocial proposers overcome their selfish impulses in both games to make a fair offer. Although possible, this alternative explanation seems less compelling as it does not offer any explanation for why a prosocial participant would decide to exert cognitive control to make a high offer and not just selfishly make a low offer in the DG.

The same reasoning applies to our findings regarding the ToM network. Potentially, prosocial individual did not engage a ToM processes in either of the games, and selfish individuals only did so in the UG, resulting in the pattern of effects we observed. This interpretation would explain why selfish participants give high offers in the UG, but it does not offer an explanation as to why prosocial players decided to give high offers in any of the games. In sum, while unlikely, we cannot rule out the alternative explanations with complete confidence; our results clearly demonstrate that not only cognitive control processes but also ToM processes differentiate between selfish and strategic players.

To conclude, we were able to dissociate prosocial from selfish players based on multivariate neural patterns during decision-making. Taking advantage of the higher sensitivity of multivariate techniques and meta-analytically defined networks associated with ToM and cognitive control, our study is the first to suggest that individual differences in prosociality are associated with differences in how cognitive control and ToM processes are engaged in the decision-making process. Our study bridges the disconnect between previous neuroimaging research focusing on the role of cognitive control and behavioral research promoting the importance of ToM in prosocial behavior. Highlighting the involvement of ToM processes in prosocial decisions, it thus contributes to a deeper understanding of the underlying neural mechanisms of human prosocial behavior. Our results suggest that in order to promote prosocial behavior in social and economic exchanges, not only fostering cognitive control capacities but also increasing the propensity to empathize and engage ToM processes is required.

## Data Availability

All data and scripts are available on figshare 10.25397/eur.11294645.
